# Fungi attacking historic wood of Fort Conger and the Peary Huts in the High Arctic

**DOI:** 10.1371/journal.pone.0246049

**Published:** 2021-01-26

**Authors:** Robert A. Blanchette, Benjamin W. Held, Joel Jurgens, Amanda Stear, Catherine Dupont

**Affiliations:** 1 Department of Plant Pathology, University of Minnesota, St. Paul, MN, United States of America; 2 Parks Canada, Nunavut Field Unit, Iqaluit, Nunavut, Canada; University of Nevada, Reno, UNITED STATES

## Abstract

Historic wooden structures in Polar Regions are being adversely affected by decay fungi and a warming climate will likely accelerate degradation. Fort Conger and the Peary Huts at Lady Franklin Bay in northern Ellesmere Island are important international heritage sites associated with early exploration in the High Arctic. Fort Conger, built by Adolphus Greely and expedition members during the First International Polar Year in 1881, was dismantled and used by Robert Peary and his expedition crew in the early 1900’s to build several smaller shelters. These historic structures remain at the site but are deteriorating. This investigation examines the fungi associated with wood decay in the historic woods. Soft rot was observed in all 125 wood samples obtained from the site. The major taxa found associated with the decayed wood were *Coniochaeta* (18%), *Phoma* (13%) *Cadophora* (12%), *Graphium* (9%), and *Penicillium* (9%) as well as many other Ascomycota that are known to cause soft rot in wood. Micromorphological observations using scanning electron microscopy of historic wooden timbers that were in ground contact revealed advanced stages of type I soft rot. No wood destroying Basidiomycota were found. Identification of the fungi associated with decay in these historic woods is a first step to better understand the unusual decomposition processes underway in this extreme environment and will aid future research to help control decay and preserve this important cultural heritage.

## Introduction

There is great concern that historic wood in Polar Regions is deteriorating and important cultural heritage will be lost [1–4]. With the Arctic experiencing a warming climate [5–7], microbial degradation is expected to increase as temperatures rise and the number of days with above freezing temperatures increase [8, 9]. As permafrost thaws, archaeological wooden remains and other organic materials are also under threat of being degraded [10–12]. Many of these archaeological materials have had extraordinary preservation by being embedded in frozen soils that have protected them for millennia. However, global warming appears to be accelerating decay rates at archaeological sites studied in Greenland [8]. Recently, the fungi associated with thawed archaeological wood from several sites ranging in age from 2500 BC to 1450 AD have been reported [9]. The major type of wood decay found in these wooden cultural properties was a soft rot produced by Ascomycota. Similar types of fungi were also found in Antarctica affecting historic huts built by Ernest Shackleton and Robert Scott during their expeditions to the South Pole in the early 1900’s [2, 13]. In both the Arctic and Antarctic, investigations strongly suggest that the fungi found attacking these archaeological and historic woods are indigenous species to these Polar Regions [2, 14–16].

**Fig 1:**
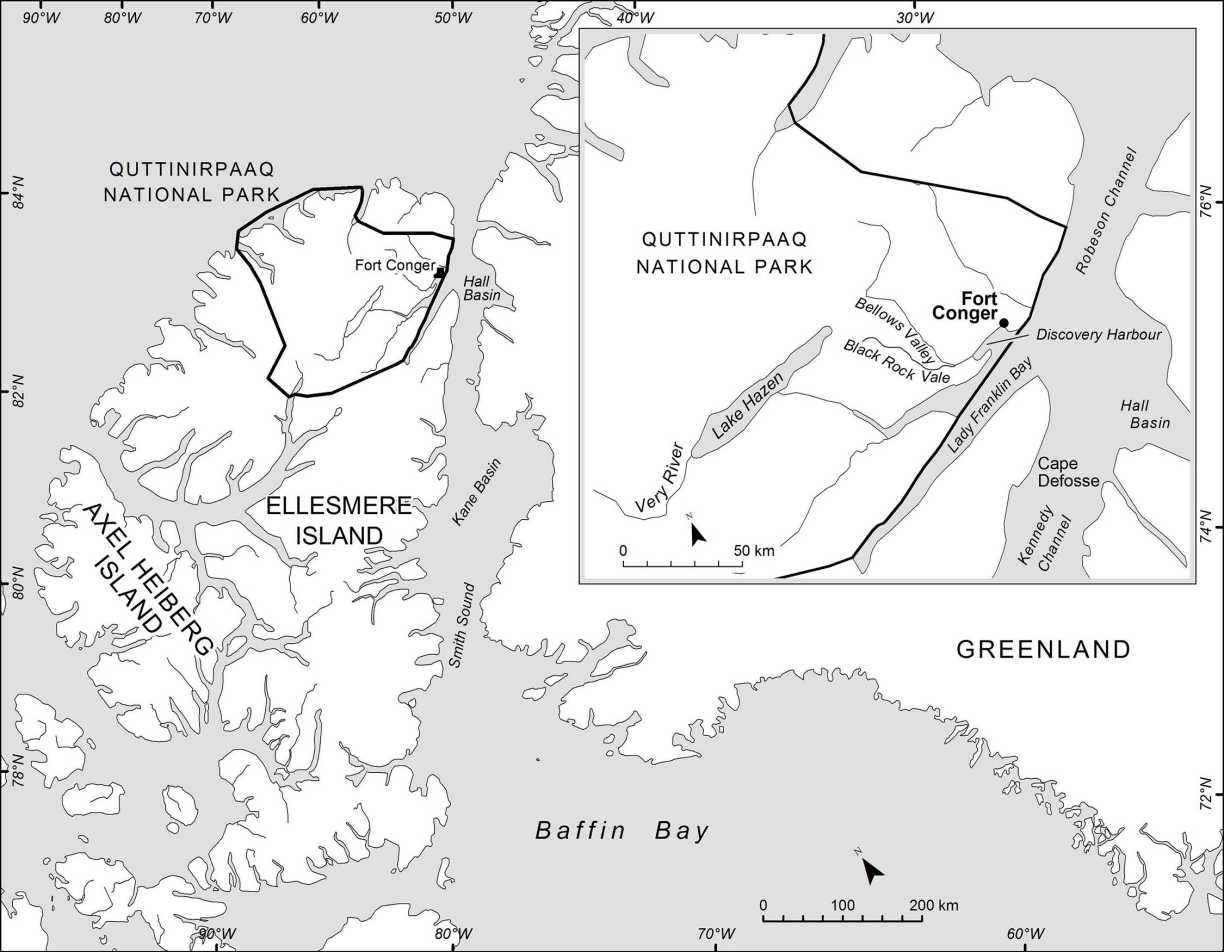
Map showing location of Fort Conger within Quttinirpaaq National Park on Northern Ellesmere Island, Nunavut, Canada.

Microbial and environmental degradation of historic huts in Antarctica has received considerable attention and conservation efforts for the Ross Sea huts by the Antarctic Heritage Trust has helped to preserve these historic structures and the cultural properties they contain [16–18]. Other sites in Antarctica, however, such as East Base on Stonington Island and wooden structures on Deception Island, are desperately in need of intervention to preserve their heritage value [3, 4, 19]. The recent investigations at multiple archaeological sites in Greenland and mummified wood in the High Arctic demonstrate that cultural heritage and ancient wood is being seriously affected by a warming climate, thawing of permafrost and accelerated microbial degradation [9, 12, 20]. Fort Conger, located at Discovery Harbour in Lady Franklin Bay on northern Ellesmere Island, Nunavut, Canada, is another very important historic site of international significance that is threatened [1, 21] (Fig 1). This location contains the remains of Fort Conger, built by A. W. Greely and US military personnel during the First International Polar Year of 1881, as well as the Peary structures built by Robert Peary and his crew as they explored the Arctic during expeditions to the North Pole.

**Fig 2:**
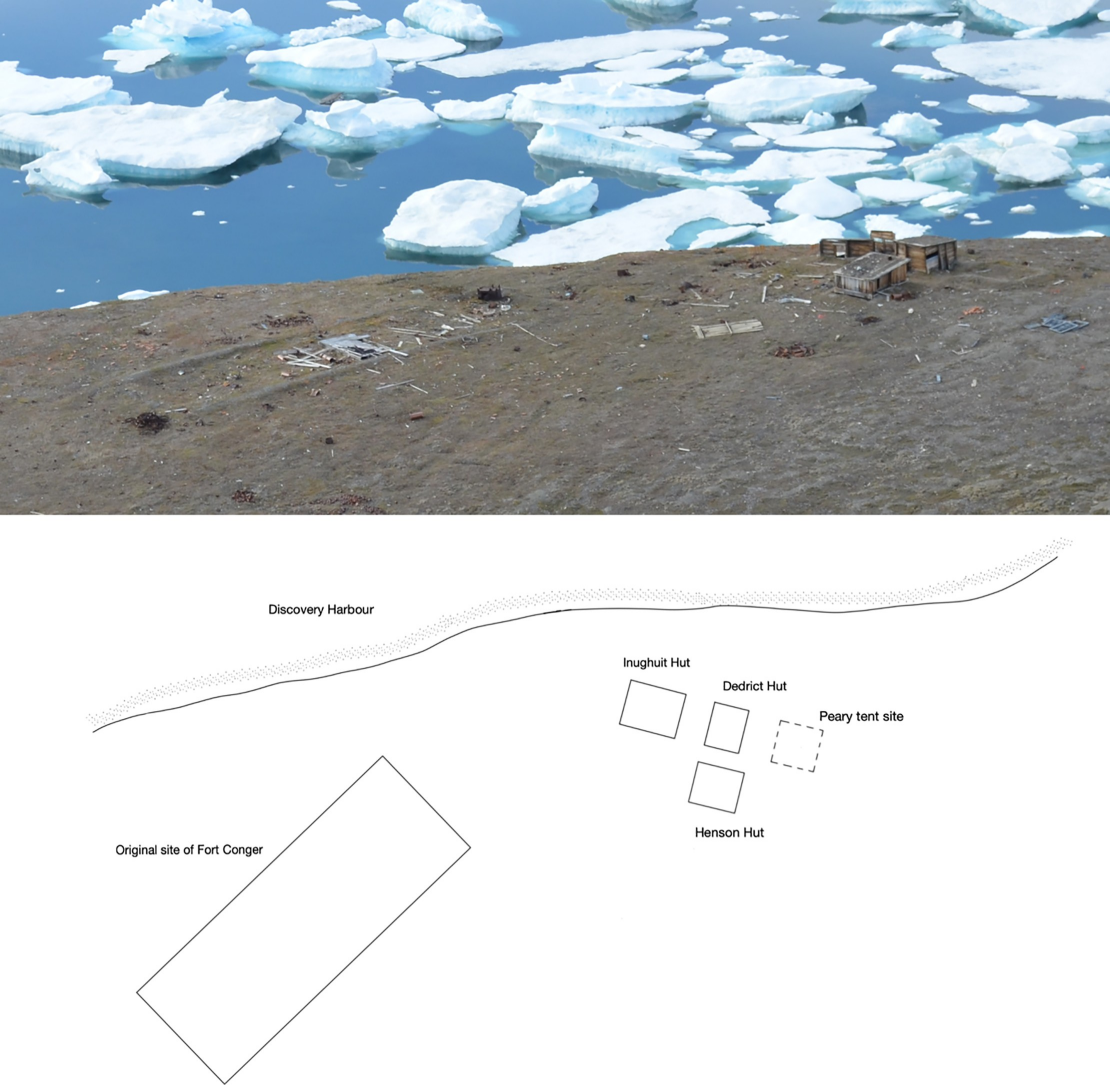
Aerial view of Fort Conger. Diagram showing the location of the original Fort built during the First International Polar Year in 1881 by Adolphus Greely with his expedition members and the three wooden shelters built during Robert Peary’s expedition in 1900. Peary dismantled Fort Conger to build the smaller huts his expedition members used. Only some floorboards and other timbers are left of Fort Conger but the Peary huts are still standing.

**Fig 3:**
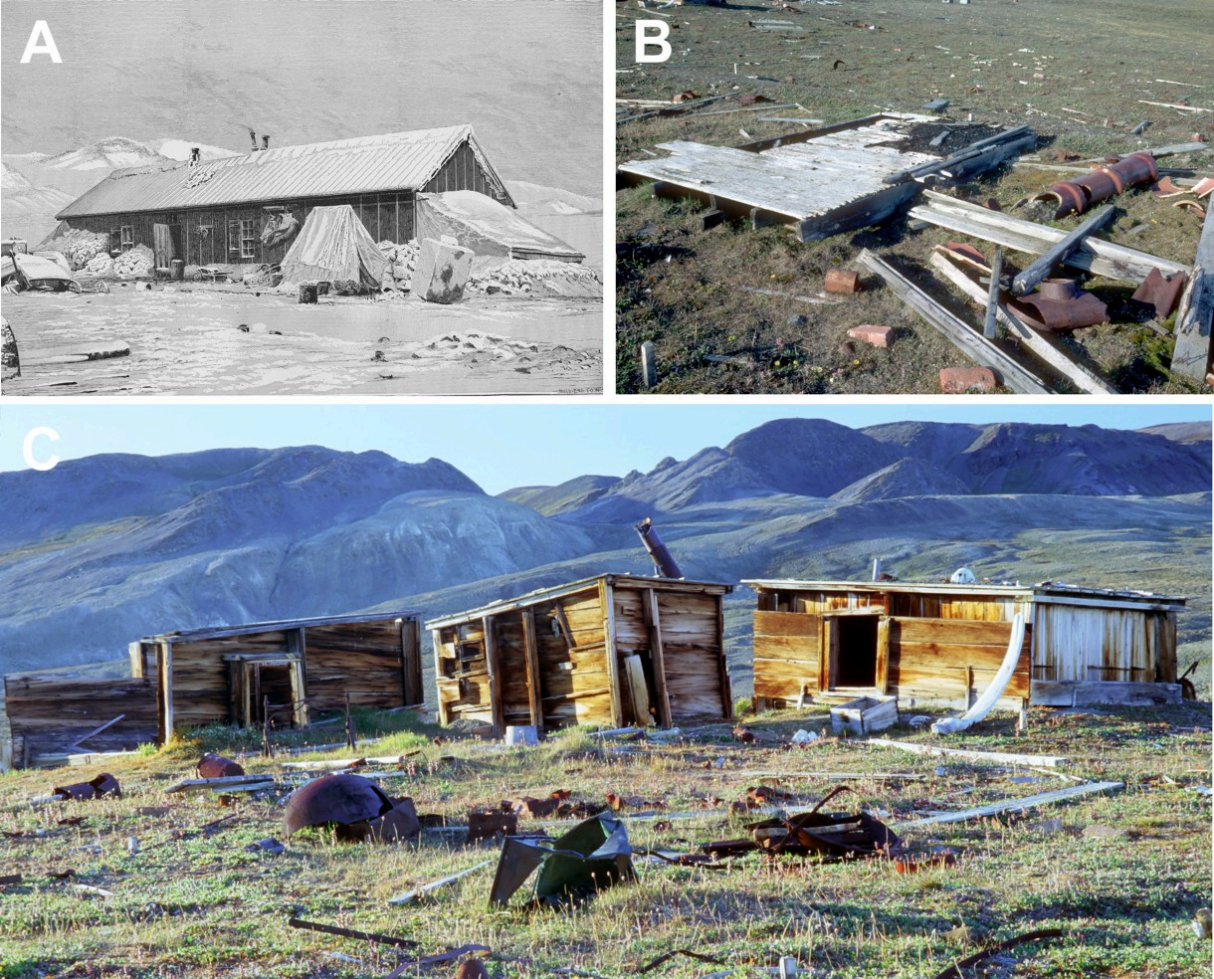
Fort Conger and huts built by Robert Peary at Lady Franklin Bay, Ellesmere Island. (A) Fort Conger as it appeared when it was built in 1881. (B) Peary and his expedition members dismantled the fort in 1900 to make smaller huts more suitable for surviving the Arctic winters. Floor boards, beams and other timbers remain at the site from Fort Conger. (C) Three wooden shelters made from the wood taken from Fort Conger and used by Peary’s expedition are still present at the site.

**Fig 4:**
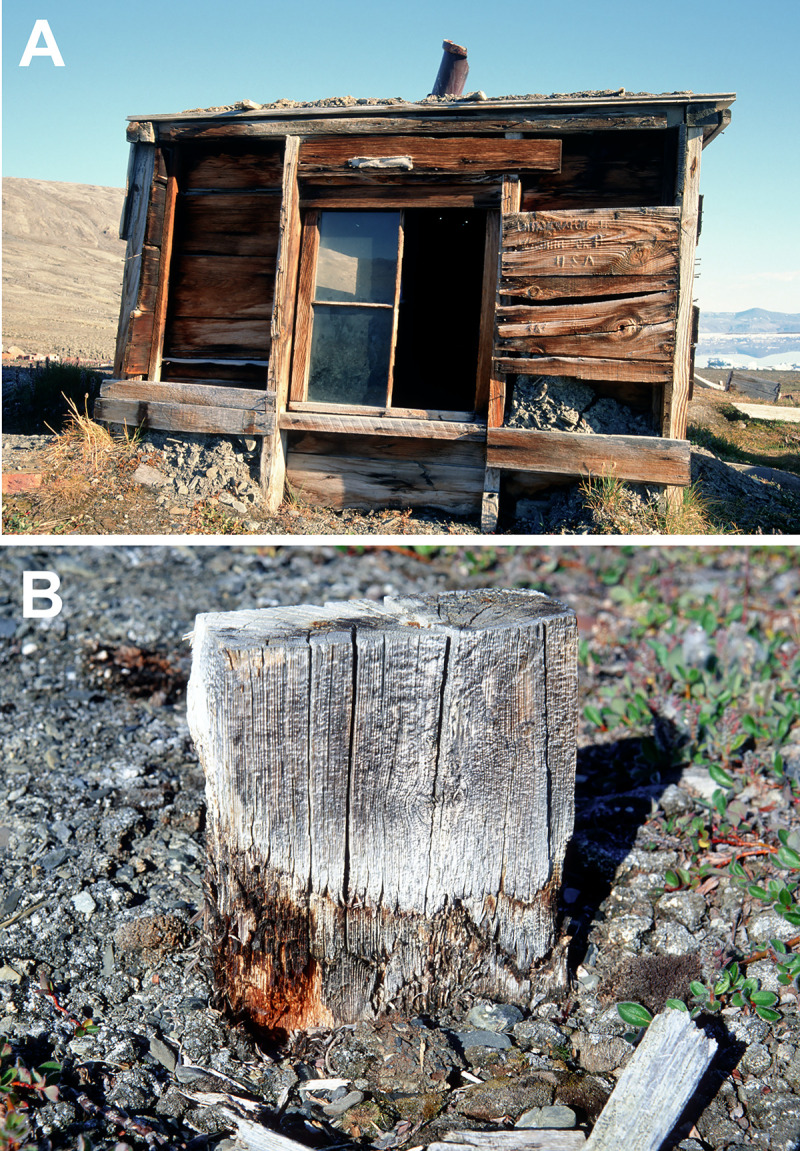
Peary expedition shelter and wood showing decay. (A) One of the huts showing it was built low into the ground and had a double wood wall that was filled with sod and soil for insulation. (B) Wood from Fort Conger in ground contact showing advanced stages of wood degradation.

Fort Conger was built as a permanent station for the United States to carry out exploration and scientific studies in the High Arctic [22]. Pre-designed and pre-fabricated the wood was transported to the site and a large wooden structure, 18 x 5.5 meters, that housed 25 men for several years was built [23] (Figs 2 and 3). In 1900, Robert Peary dismantled Fort Conger and used wood from it to build three huts using Inughuit traditional knowledge to withstand the Arctic climate [23]. These were small structures about 3 x 2 meters that were built partially into the ground (Figs 2–4). They had double wooden walls with silt and gravel packed inside as well as several other layers of insulation (Fig 4A). T.S. Dedrick and Matthew Henson, expedition members with Peary, each had their own shelter and a third hut was used by Inughuit members on the expedition. Robert Peary used a tent covered with insulating mattresses from Fort Conger and sod. The tent no longer exists at the site (Fig 2). All structures were connected by low tunnels that provided passageways from one structure to another [23]. The semi subterranean character of the dwellings worked well to provide protection for the explorers and when frozen, the wood was protected from degradation. Over time, however, warming temperatures caused thawing of the soil as well as the wood in ground contact and this moisture provided a conducive environment for decay to take place. Recent expeditions to this historical site documented biological degradation and nonbiological deterioration taking place in the wood [21, 24, 25]. In addition, environmental monitoring inside the huts over a two year period (2001 to 2003) showed an increase of hours that were above 0° C as compared to temperature data obtained in 1881–83 by the Greely expedition [21]. There was 294 and 527 more hours above 0° C, as compared to 1881–1883, recorded in the Dedrick and Henson hut respectively. Differences in temperature found in the huts were apparently due to location of the data loggers.

Wood degradation taking place in historic wood is of great concern and in order to facilitate appropriate management plans to conserve the structures, more information is needed on the type of degradation and microorganisms involved. This investigation was done to identify the fungi active in the decaying wood of the Peary Huts and Fort Conger and to provide an assessment on the decay taking place in the historic woods.

## Materials and methods

Minute wood samples approximately 1 x 2 mm were obtained from 125 locations during three events. Samples were obtained from the Peary Huts and residual floorboards and miscellaneous timbers on the ground from Fort Conger (Figs 2–4). Samples were obtained in collaboration with Parks Canada under Scientific Research License numbers 0100501 and 0301102R-M from the Nunavut Research Institute and permits QQ-01-01, QQ-02-04 and OUT-2018-28980 from Quttinirpaaq National Park. Wood samples were placed in sterile bags and kept cool until brought to the laboratory for analyses. Segments were cut from each sample using aseptic techniques and incubated on four different culture media: 1.5% Difco malt extract agar (MEA), MEA with 2 ml of lactic acid added after autoclaving, a semi-selective media for Basidiomycota that included 15 g of malt extract, 15 g of agar, 2 g of yeast extract, 0.06 g of Benlate with 0.01 g of streptomycin sulfate, and 2 ml of lactic acid added after autoclaving, and a selective media for the isolation of fungi that cause stain in wood consisting of MEA amended with 0.01 g cyclohexamide and 0.05 g chloramphenicol added after autoclaving. Ingredients for each media type were added to 1 L of deionized water. These types of media were used because of previous success obtaining diverse taxa in other investigations at terrestrial polar sites [2, 13, 26, 27]. Incubation was at 20°C to 22°C since previous studies have shown filamentous fungi from polar environments are primarily psychrotrophs or mesotrophs and can grow above 20°C [2, 9, 26, 28].

Sub-segments of wood from selected samples were also used to identify wood species and type of decay. Microscopic analysis of anatomical structures was used to identify the wood [29]. The type of decay present was determined by examination of anatomical characteristics of the decayed wood caused by the major types of wood destroying microorganisms [30–32]. Additional samples were also prepared for scanning electron microscopy using previously described methods [19, 33]. Observations were made, and images taken using a Hitachi S3500 scanning electron microscope.

DNA was isolated from pure cultures grown on malt agar (15 g malt extract, 15 g agar and 1 L deionized water) using a cetyltrimethylammonium bromide (CTAB) extraction procedure [28]. Fungal hyphae from approximately ¼ of a Petri dish were scraped from the surface of an actively growing culture and suspended in 500 ml of CTAB lysis buffer with sterile glass beads, vortexed for 1 min and centrifuged briefly to aggregate hyphal material. The internal transcribed spacer gene region (ITS) was amplified using primers ITS1F and ITS4 [34]. PCR was carried out in 25 μl reactions which contained ~12ng of DNA template, 0.25 μM forward primer, 0.25 μM reverse primer, 0.05 μg/μL BSA, 1X GoTaq® green mastermix and nuclease free sterile water. Thermocycler program parameters for amplification were: 94°C for 5 min, then 35 cycles of 94°C for 1 min, 50°C for 1 min, and 72°C for 1 min and a final extension at 72°C for 5 min. Amplicons were verified by electrophoresis on a 1% agarose gel with SYBR green 1 pre-stain and imaged with a Dark Reader DR45 (Clare Chemical Research–Denver, CO). Sanger sequencing was done with PCR primers on an ABI 3730xl DNA sequencer (Applied Biosystems–Foster City, CA). Consensus sequences were assembled using Geneious 9.0 [35] and were used with the BLASTn program [36] using the megablast option in GenBank. Identification of cultures was based on the highest BLAST match score of a genus-species accession from a taxonomic study. Sequences representative of each taxon were deposited in GenBank and given generic or higher classification.

Hobo® environmental data loggers were placed on a platform or stove above ground level inside the Dedrick and Henson huts in August 2018 and retrieved in August 2019. Temperature was recorded twice per day at 1:00am and 1:00pm. Data for 1:00 pm was tabulated to determine the number of days per year that were above 0° C. Daily temperature readings for 1881 to 1882 taken at 1:00 pm each day by the Greely expedition [22] were used to determine the number of days above 0° C at 1:00 pm for comparison to the 2018 to 2019 data.

## Results

**Fig 5:**
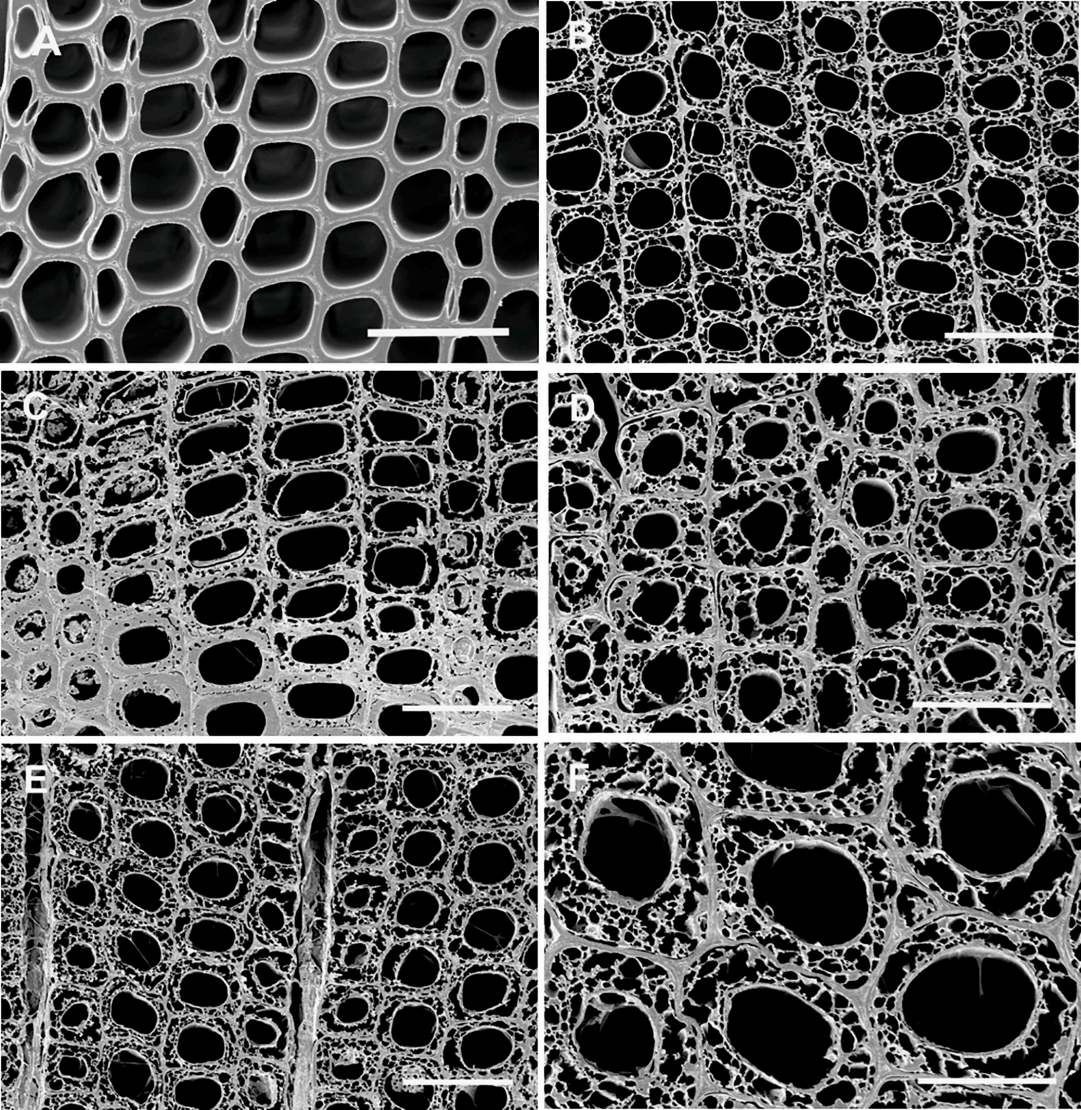
Scanning electron micrographs of transverse sections from historic woods at Fort Conger. (A) Sound pine wood showing intact tracheid cell walls. (B) Advanced stages of soft rot from Fort Conger timbers with extensive soft rot attack and cavity formation within all secondary cell walls. (C) Wood from the Inughuit hut with a progression of soft rot attack exhibiting incipient stages of attack with small individual cavities in the cell walls (bottom of photo) and more advanced stages with many coalescing cavities (top of photo). (D) Advanced decay in wood from Henson’s hut with the S2 layer of the secondary walls completely riddled with cavities and many of the cavities coalescing into lager voids. (E) and (F) Soft rot in wood from the Inughuit hut showing advanced stages of decay with secondary cell walls almost completely destroyed but the middle lamella and S3 region of the secondary wall, the wall layer closest to the cell lumen, remaining. Bar = 50μ in A to E and 25μ in F.

The wooden boards that were used to construct the Peary shelters were obtained from the dismantled Fort Conger and primarily consisted of *Pinus* species (Figs 2–4; Table 1). Both white pine and hard pine, that was likely southern yellow pine or western hard pine, were present in the hut structures in addition to a few boards made from *Betula* and *Populus* that were on the ground near the huts. Although Fort Conger was dismantled by Peary, some floorboards, beams and other timbers from Fort Conger are still present at the site (Figs 2, 3B and 4B). Additionally, sections were examined microscopically to determine the type of wood degradation that was present in the wood from Fort Conger and the Peary Huts. Out of 125 samples obtained for fungal isolations, a subset of 27 representative samples from the different structures were examined microscopically to determine the type of decay present. All woods examined had evidence of soft rot attack (Table 1). Wood in contact with the ground had extensive degradation present while wood above ground had incipient to intermediate stages of soft rot (Fig 4B). Scanning electron microscopy of representative samples clearly showed the distinct characteristics of soft rot that was observed as compared to sound unaltered wood (Fig 5A to 5F). Type I soft rot was observed in all the historic wood samples examined. This decay was characterized as having cavities present within the secondary cell walls (Fig 5B). Less advanced stages of soft rot had smaller and fewer cavities in the cell wall while wood with extensive soft rot attack had larger cavities and more of them within each secondary cell wall (Fig 5C). In advanced stages of soft rot, cavities coalesced together resulting in larger voids in the cell walls (Fig 5D). The middle lamellae between cells and the S3 layer closest to the cell lumen was not degraded in the soft rotted woods (Figs 5E and 5F). Since it is very difficult to visually quantify incipient to moderate stages of soft rot in wood and only minute samples were used in this study, quantification of the amount of decay found in these historic woods was only determined as having some soft rot or having extensive soft rot. Wood in ground contact with a soft appearance had extensive soft rot attack leaving only a residual severely damaged secondary wall (Figs 4B and 5B, 5D to 5F).

**Table 1:** Wood identification and type of decay in samples from the Peary Huts and residual wood left at Fort Conger.

Sample #	Sample Location	Wood Identification	Type of Decay
FC-1	E, on ground, Inughuit hut	*Pinus sp*. (White pine)	Extensive soft rot
FC-3	E, on ground, near Inughuit hut	*Pinus sp*. (Hard pine*)	Extensive soft rot
FC-7	NW exterior wall board, Inughuit hut	*Pinus sp*. (White pine)	Soft rot
FC-9	NW, on ground, Inughuit hut	*Betula sp*. (Birch)	Extensive soft rot
FC-11	W, exterior, at ground, Inughuit hut	*Pinus sp*. (White pine)	Soft rot
FC-13	W, exterior, at ground, Inughuit hut	*Pinus sp*. (White pine)	Extensive soft rot
FC-14	NW, interior, at ground, Inughuit hut	*Pinus sp*. (White pine)	Extensive soft rot
FC-17	Door within Inughuit hut	*Pinus sp*. (White pine)	Soft rot
FC-19	W, mid-vertical support, Inughuit hut	*Pinus sp*. (White pine)	Soft rot
FC-29	NW, vertical support, at ground, Dedrick hut	*Pinus sp*. (White pine)	Soft rot
FC-36	NE, window support, Dedrick hut	*Pinus sp*. (White pine)	Soft rot
FC-49	W, vertical support, at ground, Henson hut	*Pinus sp*. (Hard pine*)	Extensive soft rot
FC-52	S, wall, at ground, Henson hut	*Pinus sp*. (White pine)	Extensive soft rot
FC-55	S, roof board, Henson hut	*Pinus sp*. (White pine)	Soft rot
FC-56	SE, vertical support, at ground, Henson hut	*Pinus sp*. (White pine)	Extensive soft rot
FC-57	S, exterior wall, Henson hut	*Pinus sp*. (White pine)	Soft rot
FC-61	NW, tunnel wall, at ground, Henson hut	*Pinus sp*. (White pine)	Extensive soft rot
FC-63	N, tunnel door board, Henson hut	*Pinus sp*. (Hard pine*)	Extensive soft rot
FC-66	N, shutter inside Henson hut	*Pinus sp*. (White pine)	Soft rot
FC-67	NE, board in ground, Henson hut	*Pinus sp*. (White pine)	Extensive soft rot
FC-70	N, board on ground, Fort Conger	*Populus sp*.	Soft rot
FC-71	N, floor board on ground, Fort Conger	*Pinus sp*. (Hard pine*)	Extensive soft rot
FC-75	E, center floor board on ground, Fort Conger	Too degraded to I.D.	Extensive soft rot
FC-76	E, wall board on ground, Fort Conger	Too degraded to I.D.	Extensive soft rot
FC-77	Central, on ground, Fort Conger	*Pinus sp*. (Hard pine*)	Extensive soft rot
FC-79	S, floor board on ground, Fort Conger	*Pinus sp*. (Hard pine*)	Extensive soft rot
FC-83	W, wall board on ground, Fort Conger	*Pinus sp*. (Hard pine*)	Extensive soft rot

* Southern yellow pine or Western hard pine.

Culturing wood segments on a variety of different media yielded 191 pure cultures. To identify the taxa, DNA extraction of the ITS region and sequencing was used. The best Blast match of the sequence when compared to sequences in GenBank was used to identify the fungi. The match selected was the best match to a sequence that originated from a taxonomic study with 97% or greater similarity. A diverse group of Ascomycota were found (Table 2). Despite using a semi-selective media for isolating Basidiomycota, no wood destroying Basidiomycota were isolated. The most frequent genera of fungi isolated were *Coniochaeta* (18%), *Phoma* (13%) *Cadophora* (12%), *Graphium* (9%), and *Penicillium* (9%). All of these fungi were found in wood samples from each of the Peary huts as well as in wood from Fort Conger. Other Ascomycota occurred with less frequency including *Alternaria*, *Cosmospora*, *Phialophora*, *Sydowia* and *Valsa* isolated at 2 to 4%. Seven taxa were isolated just 2–4 times and twelve taxa were isolated only once from the samples collected. A good sequence match for ITS of over 97% similarity was found for 184 of the isolates when compared to sequences in GenBank. Seven of the 191 isolates obtained and sequenced with a Blast match of less than 97% (noted with an * in Table 2). All sequences for the fungi obtained from the historic woods were accessioned in GenBank (S1 Table).

**Table 2:** Fungi isolated from historic wood of Fort Conger and the Peary Huts.

Taxa	Structure and Number of Isolates				Total
	Inughuit	Dedrick	Henson	Fort Conger	
Allonectria miltina*	1				1
*Alternaria aspera*			2		2
*Alternaria atra*			1		1
*Alternaria malorum*	1				1
*Alternaria tellustris*				1	1
*Cadophora fastigiata*	1	3	1	3	8
*Cadophora luteo-olivacea*	4		1		5
*Cadophora malorum*	3	4	2	1	10
*Capronia pulcherrima*	1				1
*Cladosporium cladosporioides*		1			1
*Coniochaeta acaciae*				1	1
*Coniochaeta boothii*	2	1	1		4
*Coniochaeta cipronana* *			1		1
*Coniochaeta discospora* *			1		1
*Coniochaeta hoffmannii*	4		12	12	28
*Coniothyrium telephii*	3			1	4
*Cosmospora viridescens*	2	2	2	3	9
*Exophiala xenobiotica*		2		1	3
*Graphium silanum*	8	2	1	6	17
*Juxtiphoma eupyrena*				1	1
*Lachnum carneolum* *				1	1
*Mollisia cinerea*			1		1
*Mortierella alpine*			2		2
*Mortierella gamsii*				1	1
*Mortierella hyaline*		1			1
*Mucor hiemalis*			1	1	2
*Ochrocladosporium frigidarii*			1		1
*Oidiodendron griseum*	2		1	1	4
*Penicillium canescens*	1			1	2
*Penicillium corylophilum*	1				2
*Penicillium fimorum*	1			1	2
*Penicillium flavigenum*		1	1		2
*Penicillium glabrum*	1				1
*Penicillium samsonianum*		1			1
*Penicillium stoloniferum*	2	3			5
*Penicillium swiecickii*	1			2	3
*Phialemonium atrogriseum*	1				1
*Phialocephala lagerbergii*	1	2	1		4
*Phialophora hyalina*			2	3	5
*Phoma herbarum*	6	6	4	8	24
*Polyphilus sieberi* *		1			1
*Pseudogymnoascus pannorum*			1		1
*Purpureocillium lilacinum*	2	2			4
Pyrenopeziza ebuli*	1				1
*Sporidesmium campiniae*		1			1
*Sydowia polyspora*		2	3		5
*Tympanis piceae*				1	1
*Tympanis tsugae* *			1		1
*Valsa nivea*			1	6	7
*Xenopolyscytalum pinea*	1	2	1		4

* Indicates taxa with less than 97% similarity to the best Blast match.

Temperature readings at 1:00 pm for a one year period from August 1, 2018 to July 31, 2019 showed there were 90 days recorded above 0° C in the Dedrick hut and 93 days in Henson’s hut. Temperature data taken by Greely at the site in 1881 to 1883 at 1:00 pm indicated that there was 73 days above 0° C in 1881 to 1882 and 76 days in 1882 to 1883.

## Discussion

Analyses of the 125 wood samples from the three Peary shelters and Fort Conger timbers indicated Ascomycota were the only type of fungi present. The most frequently isolated taxa, *Coniochaeta*, *Phoma*, *Cadophora*, *Graphium*, and *Penicillium*, are known from previous investigations to cause soft rot in wood [37–40]. Other studies have found *Cadophora*, *Coniochaeta* and *Phoma* associated with soft rot in wood from polar environments [2, 4, 13, 19, 41]. During our sampling, we specifically targeted the most visually degraded wood at the site in order to obtain samples that would yield fungi responsible for the degradation as well as samples taken at random. Most of these samples were from wood in ground contact or areas of the structures above ground that appeared decayed. Therefore, our results do not reflect the overall state of preservation of the historic woods but rather focused on the most decayed areas. The foundations and other support structures in ground contact were severely decayed at the wood surfaces where samples were taken. More destructive testing to obtain samples from within the wooden timbers was not done since these are protected heritage buildings and only limited sampling at wood surfaces was authorized. Therefore, it is not known how deep into the timbers the advanced stages of soft rot attack occurred. Our results are from a culture-based study and provides information on the fungi that were in the wood at the time of sampling and apparently were actively growing.

Soft rot fungi attack the surfaces of wood and then progressively grow into the timbers [32]. There are two types of soft rot: type I causes cavities to form inside the secondary walls and is most frequently observed in the wood of conifers; type II soft rot attack causes a general cell wall erosion that degrades all of the secondary cell wall and is often found in hardwoods [32]. In both type I and type II soft rot, the middle lamellae between cells is not degraded. Soft rot fungi were also once thought to be restricted to wet environments but they are common at terrestrial sites with extreme environments that exclude other decay fungi (i.e. white and brown rot fungi). They appear to be very common in the Arctic [9, 20, 21, 28]. Soft rot in wood at terrestrial sites is often brown and cracks when dry. This can visually mimic brown rot caused by Basidiomycota. However, the micromorphology of the two types of decay are very different and soft rot does not cause a diffuse attack at a distance from the fungal hyphae and does not cause significant loss of wood strength early in the decay process as seen with brown rot. Over time, as soft rot fungi cause advanced stages of degradation with greater numbers of cavities formed in the wood cells and these cavities increasingly coalesce, the wood becomes soft and very weak. Since most of the wood in the Peary Huts and remaining Fort Conger wood is pine, we primarily found only type I soft rot. Wood exposed to this polar environment for over 100 years created appropriate conditions for advanced stages of decay to occur in many of the woods sampled.

A warmer Arctic and more time above 0°C annually allows for increased degradation to take place by these fungi. Environmental monitoring at Fort Conger indicated that warmer and longer summers are taking place as compared to monitoring that was recorded at the site by the Greely Expedition in 1881 to 1883. During Greely’s expedition, hourly temperature measurements were painstakingly taken every hour and it was possible to obtain readings taken at 1:00 pm each day for two years [22]. Although data are presented from only two data loggers for a one-year period taken each day at 1:00 pm in 2018 to 2019, we are able to make some comparisons. During this time, there were about 20 more days above 0° C as compared to the data obtained by Greely [22]. Temperature readings for the huts that were previously reported indicated that there were about 500 hours of temperatures above 0° C as compared to the number of hours reported by Greely [21]. This trend of warmer temperatures as compared to 140 years ago appears to be continuing which will allow these fungi to carry on their degradative activities and also likely accelerate their progressive attack on the historic woods. In Greenland, studies have shown that the degradation of wood is highly temperature dependent with rates increasing exponentially with increasing temperature [12]. These investigators found that over a 27 year period, soft rot fungi under freeze and thawing conditions every summer were able to degrade 25% of the dry mass of wood. Other laboratory studies have also shown that soft rot fungi, grown under conditions that are conducive for decay, can cause significant degradation (10 to 20%) after just 16 weeks of incubation [19]. Many of the soft rot fungi identified in the historic woods at Fort Conger are taxa that appear to have the ability to withstand unusual conditions. This includes tolerating repeated freeze / thawing, high concentrations of salt, high UV radiation and even high metal ion concentrations making them well suited for this High Arctic site or other extreme environments [2, 42]. Although soft rot fungi are the prevalent fungi present at the site, this could change with a continued warmer Arctic environment. In a study of archaeological wood in Greenland, some Basidiomycota were found in addition to Ascomycota [9]. Many of these Basidiomycota are wood destroying fungi that can cause brown or white rot types of decay and they can be very aggressive decomposers. The brown and white rot fungi can cause significant mass loss in a short period of time. A changing climate could influence the type of fungi colonizing polar sites and greater numbers of Basidiomycota may be found colonizing these historically important woods in the future. This increases the potential risk of more serious losses. At present, however, conditions appear restrictive to the Ascomycota that tolerate the severe polar conditions at this coastal site in the High Arctic.

Of the 191 isolates of fungi obtained from the historic wood samples, 184 had Blast matches for the ITS region of 97% similarity when compared to sequences in GenBank. Seven of the isolates had matches below 97% (Table 2, S1 Table). For these fungi that did not have a good match to known fungi, additional phylogenetic analyses and sequencing of other gene regions are needed to determine the identity of these fungi with certainty. These isolates may represent new species which is not unexpected when sampling from an environment that has received limited study.

The Ascomycota found to be the dominant taxa in the historic woods at Fort Conger appear to be indigenous fungi to the Arctic. Similar genera have been found in other studies of archaeological, historic and modern woods as well as driftwood that has been exposed to the polar environment [2, 9, 14, 28]. These fungi are likely resident populations at the site colonizing other substrates such as dead Arctic willow (*Salix* species), mosses and other plants as well as any organic matter. They appear to be opportunistic fungi and are able to colonize new carbon sources, such as the wood that had been introduced to this Arctic site during construction of Fort Conger. A good example of resident populations of indigenous fungi attacking new substrates can be seen in a study of ancient mummified, non-permineralized wood from the interior of Ellesmere Island. This wood had been released from retreating glaciers in proglacial soil and exposed to the environment. The mummified wood was found to be colonized by *Cadophora* and other soft rot fungi [20]. Research in Antarctica has also shown that fungi such as *Cadophora* that caused degradation in the historic huts built by polar explorers to the South Pole were also prevalent in soils that were sampled near the huts and even in soils located at great distances away from them [14, 26]. Genera such as *Cadophora*, *Coniochaeta*, *Mollisia*, *Phialocephala*, and *Phoma* also have been found to have endophytic relationships with plants [43–47]. These dark septate endophytes are commonly associated with plant roots in Polar Regions and appear to be widespread [48]. Their presence wherever plants grow in the Arctic could be a reservoir for these fungi allowing colonization of soils and new substrates, such as introduced wood. Currently, we have little information about the biology and ecology of these important polar fungi and their role in Arctic ecosystem functioning.

## Conclusions

Historic wood of the Peary huts and residual wood left by the Greely expedition at Fort Conger was found to be colonized by many different Ascomycota. The identity of these fungi, determined by DNA sequencing, indicates that many of these genera are known to cause a soft rot attack of wood. Micromorphological studies have shown that most of the wood samples collected had advanced stages of type 1 soft rot that consisted of cavities within secondary walls. In advanced stages, the cavities coalesce resulting in extensive degradation of the inner secondary cell walls. What remains is a fragile residue of cell wall material and wood with compromised strength properties. Environmental monitoring and temperature data collected at the site and compared to data collected by the Greely expedition 140 years ago confirms that temperatures in the Arctic have been increasing and warmer conditions are occurring at Fort Conger. With more days above 0°C, there is a longer period of time that fungi can be active and for degradation to take place. This increases the risk of losing important historic cultural heritage such as the expedition huts at Fort Conger. The ability of soft rot fungi found in the Arctic to survive extreme conditions and tolerate toxic compounds makes them difficult to manage in historic structures. Eradicating these fungi from wood exposed to the polar environment does not appear possible. Instead, controlling the environmental conditions needed for growth, such as moisture, could help limit their growth and rate of degradation. Increasing drainage around the wooden structures and allowing wood to dry out may be one of the options that conservation specialists can use to restrict fungal growth and degradation. Finding ways to keep wood frozen and prevent thawing would also restrict fungal activity. However, controlling these tenacious fungi may be difficult to accomplish. With knowledge of the fungi causing the degradation that we now have from this study, new interdisciplinary investigations can be initiated to explore and test methods to limit their growth and subsequent decay. Genomic and proteomic studies of these soft rot fungi also need to be completed to better understand their unique decomposition processes and mechanisms they utilize for saprotrophic survival in the Arctic. In a broader context, elucidating their role as key players in carbon cycling in polar ecosystems also requires research investigation.

## Supporting information

S1 Table Taxa identified from historic wood from Fort Conger and the Peary Huts with % identity to sequences in NCBI database and GenBank accession numbers. (DOCX)
