# Supplementary material for: Fungi attacking historic wood of Fort Conger and the Peary Huts in the High Arctic
Source: PLoS One. 2021 Jan 26;16(1):e0246049. doi: 10.1371/journal.pone.0246049 (PMC7837483; doi:10.1371/journal.pone.0246049)
Supplement: S1 Table — (DOCX) [file pone.0246049.s001.docx]

| **Taxa** | **% Identity** | **GenBank #** |
| --- | --- | --- |
|  |  |  |
| *Allonectria miltina** | 92 | MW033371 |
| *Alternaria aspera* | 100 | MW033332 |
| *Alternaria atra* | 100 | MW033333 |
| *Alternaria malorum* | 99 | MW033334 |
| *Alternaria tellustris* | 100 | MW033335 |
| *Cadophora fastigiata* | 100 | MW033336 |
| *Cadophora luteo-olivacea* | 100 | MW033337 |
| *Cadophora malorum* | 100 | MW033338 |
| *Capronia pulcherrima* | 98 | MW033339 |
| *Cladosporium cladosporioides* | 100 | MW033340 |
| *Coniochaeta acaciae* | 98 | MW033341 |
| *Coniochaeta boothii* | 98 | MW033342 |
| *Coniochaeta cipronana ** | 93 | MW033343 |
| *Coniochaeta discospora ** | 94 | MW033344 |
| *Coniochaeta hoffmannii* | 100 | MW033345 |
| *Coniothyrium telephii* | 100 | MW033346 |
| *Cosmospora viridescens* | 100 | MW033347 |
| *Exophiala xenobiotica* | 100 | MW033348 |
| *Graphium silanum* | 100 | MW033349 |
| *Juxtiphoma eupyrena* | 100 | MW033350 |
| *Lachnum carneolum ** | 97 | MW033351 |
| *Mollisia cinerea* | 98 | MW033352 |
| *Mortierella alpine* | 99 | MW033353 |
| *Mortierella gamsii* | 99 | MW033354 |
| *Mortierella hyaline* | 100 | MW033355 |
| *Mucor hiemalis* | 99 | MW033356 |
| *Ochrocladosporium frigidarii* | 98 | MW033357 |
| *Oidiodendron griseum* | 100 | MW033358 |
| *Penicillium canescens* | 99 | MW033359 |
| *Penicillium corylophilum* | 100 | MW033360 |
| *Penicillium fimorum* | 100 | MW033361 |
| *Penicillium flavigenum* | 99 | MW033362 |
| *Penicillium glabrum* | 100 | MW033363 |
| *Penicillium samsonianum* | 100 | MW033364 |
| *Penicillium stoloniferum* | 100 | MW033365 |
| *Penicillium swiecickii* | 100 | MW033366 |
| *Phialemonium atrogriseum* | 100 | MW033367 |
| *Phialocephala lagerbergii* | 100 | MW033368 |
| *Phialophora hyalina* | 99 | MW033369 |
| *Phoma herbarum* | 100 | MW033370 |
| *Polyphilus sieberi ** | 94 | MW033372 |
| *Pseudogymnoascus pannorum* | 100 | MW033373 |
| *Purpureocillium lilacinum* | 100 | MW033374 |
| *Pyrenopeziza ebuli** | 92 | MW033375 |
| *Sporidesmium campiniae* | 98 | MW033376 |
| *Sydowia polyspora* | 100 | MW033377 |
| *Tympanis piceae* | 98 | MW033378 |
| *Tympanis tsugae ** | 91 | MW033379 |
| *Valsa nivea* | 99 | MW033380 |
| *Xenopolyscytalum pinea* | 99 | MW033381 |
